# Why Cell Reprogramming is Functionally Linked to Aging?

**DOI:** 10.18632/aging.100364

**Published:** 2011-08-22

**Authors:** Jian Zhao, Gang Pei

**Affiliations:** ^1^ State Key Laboratory of Cell Biology, Institute of Biochemistry and Cell Biology, Shanghai Institutes for Biological Sciences, Chinese Academy of Sciences; Graduate School of the Chinese Academy of Sciences, Shanghai 200031, China; ^2^ School of Life Science and Technology, Tongji University, Shanghai 200092, P.R. China

Stem cell research meets aging research. Recent findings indicate that cell reprogramming share some regulatory mechanisms with aging. Modulation of epigenetic enzymes, blockage of TGF-β, MEK, and GSK3b cascades, as well as avoidance of oxidation can enhance the generation of iPSCs [1, 2]. Interestingly, dysfunction of these pathways has been considered as a cause of aging [3]. Our latest study adds one more evidence that inhibition of IGF/PI-3K/mTOR pathway, activation of sirtuin, induction of autophagy, which all associate with increased longevity and the delayed onset of age-related disorders [4, 5], promote somatic cell reprogramming [6].

These parallels could not be merely coincident. Organism aging involves decline of self-renew compartment function and somatic stem cell replication, which might be deteriorated by external aging-cues or aged stem cell niche [7]. Telomere dysfunction and/or DNA damage which are prevalent in aged cells correlates with a decreased reprogramming efficiency [8]. Dysregulation of signal pathways controlling cell proliferation potencies could decrease efficiency of cell reprogramming [9]. The initiation of cell reprogramming to pluripotency involves coordinated epigenetic changes that alter a genome-wide scale of chromatin structure and gene activity which is also critical for cell cycle transition. Thus reprogramming and aging also correlate well at levels of cellular function and cell signaling.

To date, somatic cell reprogramming has been achieved in vitro. It would be of great importance to explore whether the anti-aging agents, e.g. rapamycin, could function to enhance stem cell function, protect stem cell prluripotency and even promote reprogramming in vivo. It is also very interesting to verify whether some or all adult organs/tissues do possess some significant regenerative capacity due to the suspected in vivo reprogramming. Furthermore, it has been reported that agents which effectively function for a common human disease by enhancing self-renewal could lose efficacy in older individuals due to the age-associated decline of replication [10]. Thus understanding and realization of in vivo cell reprogramming is not only a fundamental theoretical question but also a very promising strategy for anti-aging and regenerative medicine.

Reprogramming of somatic cells has been enthusiastically hoped to become an arsenal to against aging as it would leads to personalized stem-cell-based rejuvenation therapies. What we learn from research of stem cell and reprogramming could help us to develop two potential anti-aging approaches in adult and older: i) to protect, ameliorate or reverse the age-associated loss function of stem cell in vivo and ii) to replace the lost stem cells by reprogrammed pluripotent cells. Considering the shared mechanisms by anti-aging and reprogramming processes, we speculate that applying a cocktail of pharmacological agents (small or macro molecules), or even the herbal extracts (such as the traditional Chinese medicines) might achieve in vivo reprogramming and eventually lead to rejuvenation.
